# Self-Esteem and Problematic Smartphone Use Among Adolescents: A Moderated Mediation Model of Depression and Interpersonal Trust

**DOI:** 10.3389/fpsyg.2019.02872

**Published:** 2019-12-20

**Authors:** Chen Li, Dong Liu, Yan Dong

**Affiliations:** ^1^Department of Psychology, Renmin University of China, Beijing, China; ^2^School of Journalism and Communication, Renmin University of China, Beijing, China

**Keywords:** problematic smartphone use, self-esteem, interpersonal trust, depression, moderated mediation model, adolescents, smartphone addiction

## Abstract

Research has found that self-esteem is negatively associated with problematic smartphone use (PSU). However, the internal mechanisms underlying that relationship need further investigation. The purpose of this study was to investigate the roles of depression and interpersonal trust in the relationship between self-esteem and PSU among adolescents. A questionnaire comprised of the Rosenberg Self-esteem Scale, Inclusive General Trust Scale (IGTS), Self-rating Depression Scale (SDS), and personal questions was administered to 637 students (female = 355) at two middle schools in Shanghai, China. Correlation analyses, mediation analysis, and moderated mediation analysis were performed. A moderated mediation model was established, which revealed: (1) a significant negative association between self-esteem and PSU, (2) depression mediated the relationship between self-esteem and PSU, and (3) the influence of depression on the relationship between self-esteem and PSU was moderated by interpersonal trust. The results indicated that low self-esteem was a risk factor, and interpersonal trust was a moderating factor for PSU among adolescents in the sample. Building adolescents’ self-esteem and increasing their interpersonal trust might decrease their PSU.

## Introduction

The past decade has witnessed a rapid rise in smartphone use. As of December 2018, the number of Chinese youth netizens exceeded 200 million, of which 90% were smartphone users (China Internet Network Information Center, 2019). Particularly among adolescents, the smartphone, as an internet terminal, is the main point of access to the internet (We Are Social and Hootsuite, 2018; China Internet Network Information Center, 2019). However, despite their portability, convenience, and versatility, smartphones are causing some problems, one of which is referred to as “problematic smartphone use” (PSU) (Kuss et al., 2018; Xie et al., 2018). PSU is defined as inappropriate or excessive uses of smartphones that might interfere with everyday life, impair social functions, and/or lead to psychological and/or behavioral problems (Billieux et al., 2015). The concept of PSU mainly derives from two similar concepts. “Problematic mobile phone use” is a term and concept found in recent literature (De-Sola et al., 2017; Jiang and Zhao, 2017) and reports published before the smartphone boom (Bianchi and Phillips, 2005; Jenaro et al., 2007; Chóliz, 2010), and “problematic internet use” or “internet addiction” is a concept that has provided theoretical and methodological support for studies on PSU (Salehan and Negahban, 2013; Lee et al., 2014; Lin et al., 2014).

Many studies have found adverse consequences of PSU, particularly for adolescents. It has a negative influence on their physical health, such as headaches (Söderqvist et al., 2008), sleep problems (Söderqvist et al., 2008; Lemola et al., 2015; Xie et al., 2018), eye and vision problems (Xie et al., 2018), and so on. Other studies found that PSU damaged adolescents’ psychological and social functions (Yen et al., 2009), and it led to behavioral problems, such as aggression and smoking (Augner and Hacker, 2012). Furthermore, PSU might be associated with concentration difficulties (Söderqvist et al., 2008) and poor academic performance (Hawi and Samaha, 2016; Samaha and Hawi, 2016; Yang et al., 2019).

Because PSU might impede adolescents’ healthy development, concerns are growing about the possible correlates of PSU, such as various features of personality, emotion, interpersonal relationships, and so on (Bianchi and Phillips, 2005; Butt and Phillips, 2008; De-Sola et al., 2017; Wang et al., 2017; Kim and Koh, 2018). Some scholars proposed that PSU is an addiction-like phenomenon, similar to problem gambling (Billieux et al., 2015; Wang et al., 2016; Elhai and Contractor, 2018). Therefore, many studies have adopted the behavioral addiction perspective (Jiang and Zhao, 2017; Ihm, 2018). Because adolescents are in a critical period of physical and psychological development, exploring the causes of PSU helps us better mitigate smartphones’ adverse consequences.

Self-esteem deserves attention as a possible cause. Self-esteem is about mental representations of the self-regarding overall feelings of self-worth and self-acceptance (Rosenberg, 1965), and self-esteem is essential to adolescents’ social development and social adaptation (Robins and Trzesniewski, 2005; Trzesniewski et al., 2006). Based on the ecological systems theory and dual-factor resiliency theory, an explanatory model of adolescent problem behavior recognizes low self-esteem as one of the individual-level risk factors (Jessor et al., 2003). Many previous studies have found robust associations between low self-esteem and behavioral problems or deviance, and it has been related to aggression (Donnellan et al., 2005; Garofalo et al., 2016), smoking (Carters and Byrne, 2013), alcohol and/or drug use (Wild et al., 2004; Kavas, 2009), problem gambling (Rodda et al., 2004), and delinquency (Donnellan et al., 2005). Meanwhile, a cognitive-behavioral model of internet addiction also suggests that low self-esteem is a critical aspect of maladaptive cognition of the self, which is the core factor leading to problematic internet use (Davis, 2001). Of particular relevance to the current study, research has linked self-esteem and PSU such that low self-esteem is a risk factor of PSU (Butt and Phillips, 2008; Ha et al., 2008; Billieux, 2012; Isiklar et al., 2013), and self-esteem often has been identified as an antecedent of PSU. Wang et al. (2017) suggested that low-quality peer relationships among adolescents might lead to low self-esteem, which, in turn, might lead to PSU. Kim and Koh (2018) also found that low self-esteem was associated with PSU.

Although many studies have found direct relationships between self-esteem and PSU, more research about the internal mechanisms is needed. First, a systematic review pointed out that the link between self-esteem and PSU found by previous studies is inconsistent, such that the bivariate effects were small or moderately strong and the effects found in multivariate analyses were weaker or non-significant (Elhai et al., 2017). Therefore, exploring mediating and moderating factors might shed light on the relationship. In addition, a better understanding of mediating and moderating influences might improve our understanding of the etiology of PSU and support development of effective interventions.

Davis’s (2001) cognitive-behavioral model proposes that psychological distress, such as depression, is an essential and significant catalyst of problematic internet use. Recent studies on PSU indicated that depression predicted PSU (Panova and Lleras, 2016; Kuss et al., 2018), and it has been linked to problematic internet use, problem gambling, and other behavioral problems (Armstrong et al., 2000; Rodda et al., 2004). Kim et al. (2015) suggested that, when individuals felt depressed, they tend to use their smartphones to cope with their negative emotions. In other words, using a smartphone is considered an experiential avoidance strategy to divert aversive emotional content. However, experiential avoidance is ineffective for achieving that outcome, and, instead, it has adverse consequences (Kim et al., 2015; Chou et al., 2018). Meanwhile, influential theories of depression have considered aspects of low self-esteem as a vulnerability factor that confers risk to depression. For instance, Orth and Robins (2013) validated the vulnerability model of the relationship between low self-esteem and depression, which considered self-esteem to be a leading cause of depression. Further, individuals with lower self-esteem was more likely to develop depression than those with high self-esteem (Orth et al., 2014). From the view of the buffer hypothesis, low self-esteem is also one of the most important susceptibility factors to depression, which tends to lead to depression in individuals under the influence of stressful life events (Murrell et al., 1991; Hankin et al., 2007). In particular, the hopelessness and self-esteem theory of depression holds that depression occurs as a result of negative attributional style, low self-esteem and negative life events, where hopelessness plays a mediating role, low self-esteem playing a stimulating role, and high self-esteem acting as a buffer (Metalsky et al., 1993). A meta-analysis of longitudinal studies supported this contention, revealing that the effect of self-esteem on depression was significantly stronger than that of depression on self-esteem (Sowislo and Orth, 2013). This implies that depression might play a mediating role in the relationship between self-esteem and PSU.

Interpersonal trust is vital to healthy psychosocial development and key to the formation and maintenance of healthy interpersonal relationships (Gurtman, 1992; Simpson, 2007). Interpersonal trust has been defined as a psychological state of voluntarily placing oneself in an undefended or vulnerable position based on confident expectations of the good intentions and actions of others (Rousseau et al., 1998; Haselhuhn et al., 2015). The emancipation theory of trust proposes that people with high levels of interpersonal trust often expand their social networks (Yamagishi and Yamagishi, 1994; Yamagishi, 1998). Previous studies also found that interpersonal trust among children and adolescents related to higher social status (Buzzelli, 1988), less loneliness (Rotenberg et al., 2004), and better-quality peer relationships (Rotenberg et al., 2004). Ihm (2018) interpreted PSU as a social problem stemming from a lack of offline social networks and in-person social support. When an individual’s needs are not being met, she or he might turn to virtual sources of support as an alternative, which might lead to pathological smartphone usage. Davis’s (2001) cognitive-behavioral model also proposes that the need for social contact and reinforcement obtained online would increase the desire to remain in a virtual social life. From this perspective, the benefits of interpersonal trust mentioned above might reduce the emergence of PSU by providing alternative ways to meet individuals’ needs.

Previous studies have also found a negative association between trust and depression (Kim et al., 2012; Betts et al., 2017). Specifically, lower interpersonal trust increased the incidence of depression (Kim et al., 2012; Frank et al., 2014; Betts et al., 2017), whereas higher interpersonal trust (as a component of social capital) buffered the effects of financial stress on depression (Frank et al., 2014). Moreover, individuals with higher interpersonal trust had better psychosocial adjustment (Lester and Gatto, 1990; Rotenberg et al., 2004). A study of Chinese and American adolescents and young adults found that interpersonal trust facilitated the support-seeking process and was associated with appropriate help-seeking, which, in turn, predicted the likelihood of expressing emotional distress to friends (Mortenson, 2009). In this way, trust might also mitigate the adverse effects of depression. Therefore, interpersonal trust might buffer the relationship between self-esteem and PSU via depression.

This study’s main objectives were to explore the relationships among self-esteem, depression, interpersonal trust, and PSU and to test a moderated mediation model of the influence of self-esteem on PSU mediated by depression and moderated by interpersonal trust. Based on the results of previous studies, three hypotheses were proposed as follows.

Hypothesis 1. Self-esteem would be negatively related to problematic smartphone use.

Hypothesis 2. Depression would mediate the effect of self-esteem on problematic smartphone use.

Hypothesis 3. Interpersonal trust would moderate the direct and indirect relationships between self-esteem and PSU via depression. Specifically, interpersonal trust would buffer the direct effect of self-esteem on problematic smartphone use (Hypothesis 3a), and buffer the mediating influence of depression on the effect of self-esteem on problematic smartphone use (Hypothesis 3b).

## Materials and Methods

### Participants and Procedures

The data for this study were collected by trained and experienced research assistants in the classrooms of two middle schools in Shanghai, China, in May 2016. The respondents completed an anonymous questionnaire comprising several self-report inventories and personal questions. A token gift notebook was given to each respondent. Initially, 689 adolescents were asked to participate. Of them, 52 did not use a smartphone or did not answer all the questions, and they were dropped from the analysis. The valid sample used in the analysis comprised 637 respondents (92% response rate) (*M*_age_ = 15.38 years, *SD* = 1.29 years), of which 355 (55.7%) were female.

### Measures

#### PSU

Problematic smartphone use was measured by a 33-item inventory developed by Xie et al. (2018). This inventory covers four aspects of PSU: overuse, withdrawal, compulsive behavior, and disturbances. The items describe problems, obsession, or dysfunction related to smartphone use, such as “I feel restless and irritable when the smartphone is unavailable” and “My grades or school work suffer because of overuse of the smartphone.” Adolescents were asked to report how often they experienced these problems on a four-point Likert-type scale where 1 = *never* through 4 = *always*. The scale demonstrated strong internal reliability in the current study (α = 0.95).

#### Self-Esteem

The Rosenberg Self-Esteem Scale (Rosenberg, 1965) was used to measure self-esteem. The scale includes 10 items (e.g., “I am able to do things as well as most other people”). The respondents rated each item on a five-point Likert-type scale where 1 = *not very true of me* through 4 = *very true of me*, with higher scores representing higher self-esteem. Cronbach’s α of the scale in this study was 0.82.

#### Depression

Depression was assessed by the Self-rating Depression Scale (SDS; Zung, 1965), which consists of 20 items [e.g., “I feel down hearted and blue” and “I feel hopeful about the future” (reverse-coded)]. Each item was scored on a four-point scale where 1 = *never* through 4 = *always* and higher scores indicated higher depression. In the current study, Cronbach’s α for the SDS was 0.79.

#### Interpersonal Trust

Interpersonal trust was measured using the Inclusive General Trust Scale (IGTS), a nine-item scale developed by Yamagishi et al. (2015). This scale captures the belief aspect (e.g., “Generally, I trust others”) and the preference aspect (e.g., “I hate to lose because of having counted on someone” [reverse-coded]) of trust. Cronbach’s α for the IGTS in the current study was 0.80.

### Procedure

First, the research assistants explained the study and the questionnaire to the prospective respondents in the classrooms. Second, the respondents gave their written informed consent. Third, the questionnaire was distributed, and the adolescents completed it. The research assistants remained in the classrooms to answer any questions that they might have had. It took about 15 min for all of the adolescents to complete the questionnaire, which was collected at that time. Last, the respondents were debriefed and received their token of appreciation for participating in the study.

### Statistical Analyses

The analyses were performed using IBM SPSS software. The mediation model (Model 4) and the moderated mediation model (Model 59) were tested using the PROCESS macro (Hayes, 2013). Age and gender were included in both models as control variables. The indirect effects were tested with bias-corrected bootstrapping (*n* = 5,000) and 95% confidence intervals (CI) for the indices. When a 95% bootstrapped CI does not include zero, it indicates the parameter is statistically significant.

### Ethics

This study was conducted with the approval of the Research Ethics Review Committee of the author’s institution. All participants were well informed in advance and debriefed at the end.

## Results

### Descriptive Statistics

Means, standard deviations, and Pearson’s correlations (*r*) were calculated on all the study variables (Table 1). As expected, self-esteem and PSU were negatively correlated, depression negatively correlated with self-esteem, and depression positively correlated with PSU. Interpersonal trust positively correlated with self-esteem, and it was negatively correlated with PSU and with depression. All bivariate correlations were statistically significant (*p* < 0.001). No independent samples *t*-test results on the gender differences in the variables were statistically significant.

**TABLE 1:** Descriptive statistics and correlations among variables.

	***M***	***SD***	**1**	**2**	**3**	**4**
1. PSU	1.76	0.46	1			
2. SE	2.80	0.46	–0.21***	1		
3. DEP	1.93	0.41	0.31***	–0.62***	1	
4. IT	3.39	0.81	–0.14***	0.34***	–0.47***	1
5. Age	15.38	1.29	0.05	0.11**	–0.14***	0.15***

*N* = 637. PSU, problematic smartphone use; SE, self-esteem; DEP, depression; IT, interpersonal trust. **p < 0.01; ***p < 0.001.

### Mediating Effect of Depression

Hypothesis 1 and 2 was tested controlling for the effects of age and gender (Table 2). The total effects of self-esteem on PSU were statistically significant (β = −0.22, SE = 0.04, *p* < 0.001), indicating that the respondents with lower self-esteem had higher PSU, which supported Hypothesis 1. Although self-esteem had no direct effect on PSU, it directly influenced depression, and the association of depression with PSU also was significant. The standardized indirect effect of self-esteem on PSU via depression was significant, indirect effect = −0.18, SE = 0.03, 95% CI = [−0.24, −0.12], and the indirect effect’s proportion of the total effect was 82.7%. In support of Hypothesis 2, depression had a mediating effect on the relationship of self-esteem to PSU through its negative relationship with self-esteem (depression increased as self-esteem decreased), which, in turn, related to higher PSU.

**TABLE 2:** Testing the mediating effect of self-esteem on PSU.

**Predictors**	**On DEP**				**On PSU**			
	**β**	**SE**	***p***	**95% CI**	**β**	**SE**	***p***	**95% CI**
Gender	0.03	0.03	0.87	[−0.03, 0.09]	0.05	0.04	0.16	[−0.02, 0.13]
Age	–0.07	0.03	0.02	[−0.14, −0.01]	0.07	0.04	0.06	[−0.002, 0.15]
SE	–0.61	0.03	<0.001	[−0.67, −0.55]	–0.04	0.05	0.43	[−0.13, 0.06]
DEP					0.30	0.05	<0.001	[0.20, 0.39]
*R*^2^	0.39		<0.001		0.05		<0.001	
*F*	133.83				11.86			

*Analyses conducted using PROCESS model 4, *N* = 637. Gender was dummy coded (1 = female and 0 = male). SE, self-esteem; DEP, depression; PSU, problematic smartphone use.*

### Moderated Mediation Effects

Hypotheses 3a and 3b were tested by estimating a moderated mediation model (model 59) with PROCESS macro (Hayes, 2013) that included age and gender as control variables.

Table 3 indicates there was a significant negative direct influence of self-esteem on depression; however, its direct effect on PSU was not statistically significant. A significant moderating effect of interpersonal trust on the direct effect of self-esteem on PSU was found (Table 3). We plotted the results for PSU predicted by self-esteem separately for low (one standard deviation below the mean) and high (one standard deviation above the mean) interpersonal trust (Supplementary Figure 1). Simple slope tests revealed that respondents with high trust and relatively high self-esteem had significantly lower PSU, β_simple_ = −0.16, SE = 0.07, *p* = 0.02, 95% CI = [−0.30, −0.02]. However, for the respondents with low trust, the direct effect of self-esteem on PSU was not statistically significant, β_simple_ = 0.05, SE = 0.06, *p* = 0.43, 95% CI = [−0.07, 0.17].

**TABLE 3:** Testing the moderated mediating effect of self-esteem on PSU.

**Predictors**	**On DEP**				**On PSU**			
	**β**	**SE**	***p***	**95% CI**	**β**	**SE**	***p***	**95% CI**
Gender	0.03	0.03	0.32	[−0.03, 0.09]	0.05	0.04	0.20	[−0.03, 0.12]
Age	–0.04	0.03	0.16	[−0.10, 0.02]	0.10	0.04	0.008	[0.03, 0.18]
SE	–0.51	0.03	<0.001	[−0.58, −0.45]	–0.06	0.05	0.24	[−0.15, 0.04]
SE × IT	<0.001	0.03	1.00	[−0.05, 0.05]	–0.11	0.05	0.02	[−0.20, −0.01]
DEP					0.27	0.05	<0.001	[0.17, 0.37]
DEP × IT					–0.11	0.05	0.01	[−0.20, −0.03]
*R*^2^	0.46		<0.001		0.12		<0.001	
*F*	108.23				11.89			

*Analyses conducted using PROCESS model 59, *N* = 637. Gender was dummy coded (1 = female and 0 = male). SE, self-esteem; IT, interpersonal trust; DEP, depression; PSU, problematic smartphone use.*

The direct effect of depression on PSU was statistically significant (Table 2). However, the relationship between depression and PSU was moderated by interpersonal trust. Simple slope tests indicated that the association of depression with PSU was weaker for respondents with high trust (i.e., one standard deviation above the mean; β_simple_ = 0.16, *p* = 0.03, 95% CI = [0.01, 0.30]) than for respondents with low trust (i.e., one standard deviation below the mean; β_simple_ = 0.39, SE = 0.06, *p* < 0.001, 95% CI = [0.26, 0.51]; Supplementary Figure 2).

**FIGURE 1:**
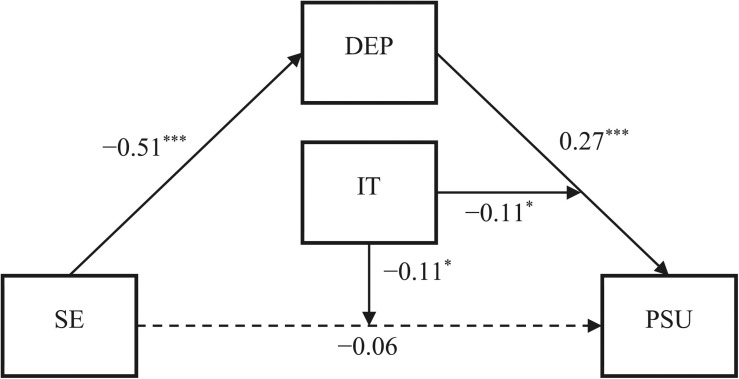
The moderated mediation model controlling for age and gender (all coefficients standardized). SE, self-esteem; DEP, depression; PSU, problematic smartphone use; IT, interpersonal trust. *p < 0.05; ***p < 0.001.

Last, the bias-corrected percentile bootstrap method further revealed a significant moderated mediation effect, β = 0.12, SE = 0.05, 95% CI = [0.02, 0.22], in which interpersonal trust moderated the mediating effect of depression by buffering its influence on PSU. The indirect effect of self-esteem on PSU via depression was statistically significant for the respondents with low trust (i.e., one standard below the mean), β = −0.20, SE = 0.04, 95% CI = [−0.27, −0.13]. In contrast, this indirect effect was non-significant for respondents with high trust (i.e., one standard above the mean), β = −0.08, SE = 0.04, 95% CI = [−0.16, 0.0001]. The moderated mediation model is shown in Figure 1. Given that interpersonal trust moderated just the second stage of the mediation process, a second-stage moderation model, which is a type of moderated mediation model, was established (Hayes, 2013). Therefore, Hypothesis 3 was partially supported.

## Discussion

Empirical support exists for a link between self-esteem and PSU in which self-esteem negatively predicts PSU. However, its internal mechanisms are ambiguous. The purpose of this study was to investigate whether self-esteem indirectly related to PSU via depression and whether the direct and indirect associations between self-esteem and PSU were moderated by interpersonal trust. The findings indicated that the predictive effect of low self-esteem on PSU was mostly explained by increased depression, and it was buffered by interpersonal trust. For respondents with high trust, higher self-esteem was associated with less PSU; however, for respondents with low trust, the direct effect of self-esteem on PSU was not significant. Further, the association between depression and PSU was weaker for the high trust rather than the low trust respondents. Thus, these analyses established a moderated mediation model.

### Relationship Between Self-Esteem and PSU

We found a significant total effect of self-esteem on PSU, which supported the findings of previous studies (Butt and Phillips, 2008; Ha et al., 2008; Billieux, 2012; Isiklar et al., 2013). Davis’s (2001) cognitive-behavioral model proposes that maladaptive cognition of the self is the core factor leading to problematic internet use, and low self-esteem certainly is a critical aspect of maladaptive cognition. Individuals with low self-esteem might believe that they are held in high regard only in online interactions, and, consequently, they use smartphones to obtain approval and recognition from others. Further, according to the terror management theory of self-esteem (Greenberg et al., 1997), when individuals encounter threats to the development of self-esteem, their need for self-protection is compensated through particular channels. In this way, problem behaviors, including PSU, seem to be compensatory. With the help of various smartphone functions, individuals might meet their needs for esteem through channels not available in the physical world, which, in turn, creates dependence on smartphones.

### The Mediating Role of Depression

We developed a mediation model of depression to examine the indirect influence between self-esteem and PSU. As hypothesized, depression mediated the influence of self-esteem on PSU; however, the direct association between self-esteem and PSU was not statistically significant. This finding might be because of the relatively strong link we found between self-esteem and depression, which supports previous studies (Smetaniuk, 2014; Elhai et al., 2017). Because no other intermediate variables were analyzed, other factors in this indirect path of depression could not be compared. In the current study, depression largely explained the predictive effect of self-esteem on PSU, which supports previous literature (Elhai et al., 2017).

This study again supports the hopelessness and self-esteem theory and the vulnerability model of depression that low self-esteem affects depression (Metalsky et al., 1993; Orth and Robins, 2013). From this perspective, life events can undermine the individual’s psychological protection system through the loss of self-esteem, leading to depression. This study also supports the affective component of PSU causes, that depression plays an important role in the occurrence of PSU.

### The Moderating Role of Interpersonal Trust

Interpersonal trust is prominent in theoretical models of lifelong development; its importance has become self-evident. However, few previous studies have analyzed the role of interpersonal trust in the development of PSU or related behavioral problems. Our study identified a second-stage moderation model regarding interpersonal trust and PSU, which enriches the literature of relevant fields. The current study’s adolescent respondents with high interpersonal trust reported relatively good psychosocial adjustment, which aligns with previous studies’ results (Lester and Gatto, 1990; Rotenberg et al., 2004). Specifically, the buffering effect of interpersonal trust suggests two dimensions. First, according to the emancipation theory of trust (Yamagishi and Yamagishi, 1994; Yamagishi, 1998), individuals with relatively high trust have relatively stable social networks and more social support, which mitigate the negative effects of low self-evaluations and negative emotions. Second, individuals with high interpersonal trust have people they trust to confide in when they feel depressed, which, in turn, reduces the likelihood of PSU.

However, interpersonal trust did not moderate the direct relationship between self-esteem and depression. This was probably because low self-esteem was directly related to increased depression. In a near-infrared spectroscopy study of social pain, Yanagisawa et al. (2011) proposed that general trust and self-esteem, as two psychosocial resources, function at different times during a series of adaptive processes, which reminds us that trust and self-esteem might separately influence depression. Further research is needed to understand the underlying reasons for such findings.

### Limitations and Implications

Several limitations should be noted when interpreting this study’s findings. First, all of the measures were self-reports, which might have influenced the study’s validity. Future studies could employ other methods, such as measuring self-esteem using the implicit association test (e.g., Greenwald and Farnham, 2000), collecting objective behavioral data on PSU (e.g., Elhai and Contractor, 2018), and applying other-report measures to reduce potential common method bias. Second, the study design was cross-sectional, eliminating the possibility of causal inferences (West, 2011). Longitudinal designs should be used for future studies to explore change in the relevant factors and establish temporal order. Third, variables on online activities might suggest interpretations from a different perspective. Future studies that examine the influences of variables, such as online interpersonal trust, might further our understanding of the similarities in and differences between online and in-person social relations/interactions (e.g., Young and Tseng, 2008).

Limitations aside, this study extends Davis’s (2001) model to the PSU field and contributes to the understanding of the etiology of PSU and behavioral addiction. This study highlights the affective component of PSU causes and takes interpersonal trust into account, enriching the ecological model of PSU. Furthermore, the findings of this study could help to guide targeted interventions for PSU in adolescents. First, in the family and school education practice, given the characteristics of adolescent self-esteem development, attention should be paid to enhancing and maintaining self-esteem, such as by attribution training (Metalsky et al., 1993), group counseling (Mackeen and Herman, 1974), and family based intervention (Danielsen et al., 2013), thereby reducing the susceptibility to depression, and thus reducing PSU. Second, given the role of interpersonal trust, establishing sound social networks and positive interpersonal interaction, especially peer interaction, thereby increasing interpersonal trust, would also reduce the emergence of PSU. Social cognitive training (Barrett et al., 1999) would help in this way. Third, on such an issue of PSU, not only the cognitive issues should be taken into consideration, but also the affective issues. Effective social support and psychological guidance would also help adolescents to use new technologies rationally and properly.

## Conclusion

This study tested a moderated mediation model to examine the psychological factors underlying the relationship between self-esteem and PSU. In brief, the results found that low self-esteem was a risk factor for problematic use of smartphones in a sample of Chinese middle school students, and it predicted PSU through the level of depression, the effects of which on PSU were buffered by interpersonal trust. These findings substantially contribute to our understanding of PSU and behavioral addiction.

## Data Availability Statement

The raw data supporting the conclusions of this article will be made available by the authors, without undue reservation, to any qualified researcher.

## Ethics Statement

The studies involving human participants were reviewed and approved by the Research Ethics Committee of Renmin University of China. Written informed consent to participate in this study was provided by the participants’ legal guardian/next of kin.

## Author Contributions

CL and YD analyzed the data and wrote the draft. DL and YD collected the data, supervised the study, revised the manuscript, and provided the funding sources. All authors designed the study, had full access to all the data, and have taken responsibility for the integrity of the data and accuracy of the data analysis.

## Conflict of Interest

The authors declare that the research was conducted in the absence of any commercial or financial relationships that could be construed as a potential conflict of interest.
