# Supplementary material for: Self-Esteem and Problematic Smartphone Use Among Adolescents: A Moderated Mediation Model of Depression and Interpersonal Trust
Source: Front Psychol. 2019 Dec 20;10:2872. doi: 10.3389/fpsyg.2019.02872 (PMC6933501; doi:10.3389/fpsyg.2019.02872)
Supplement: Supplementary file 1 [file Table_1.pdf]

Supplementary Material

1 Supplementary Figures

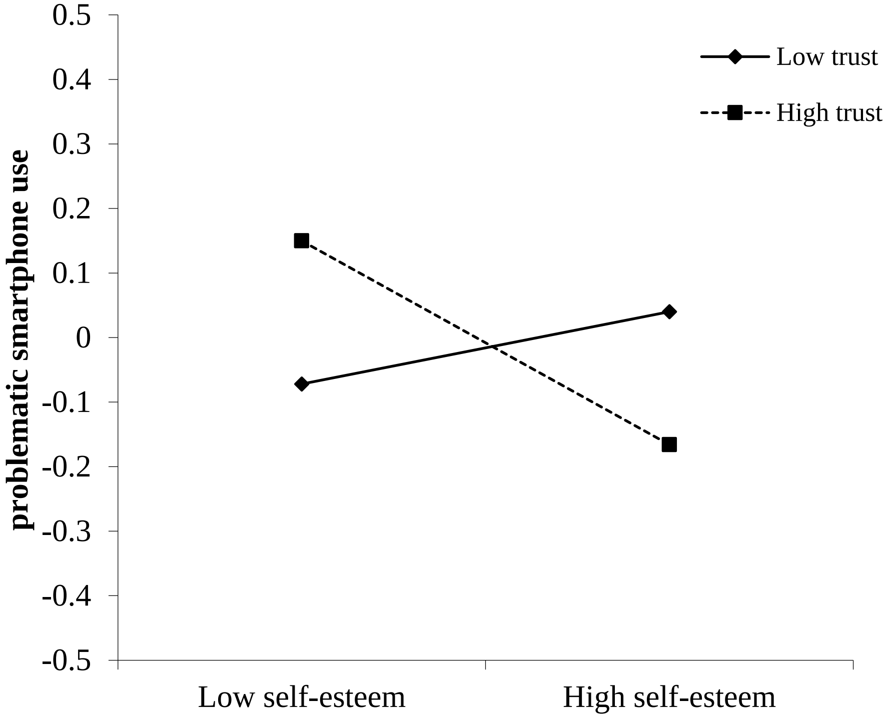

Supplementary Figure 1. Problematic smartphone use (Z-score) as a function of self-esteem and interpersonal trust. Functions are graphed for two levels of interpersonal trust: one standard deviation above the mean and one standard deviation below the mean. Note that the graph is for descriptive purpose only. All inferential analyses maintained the continuous values of self-esteem and trust.

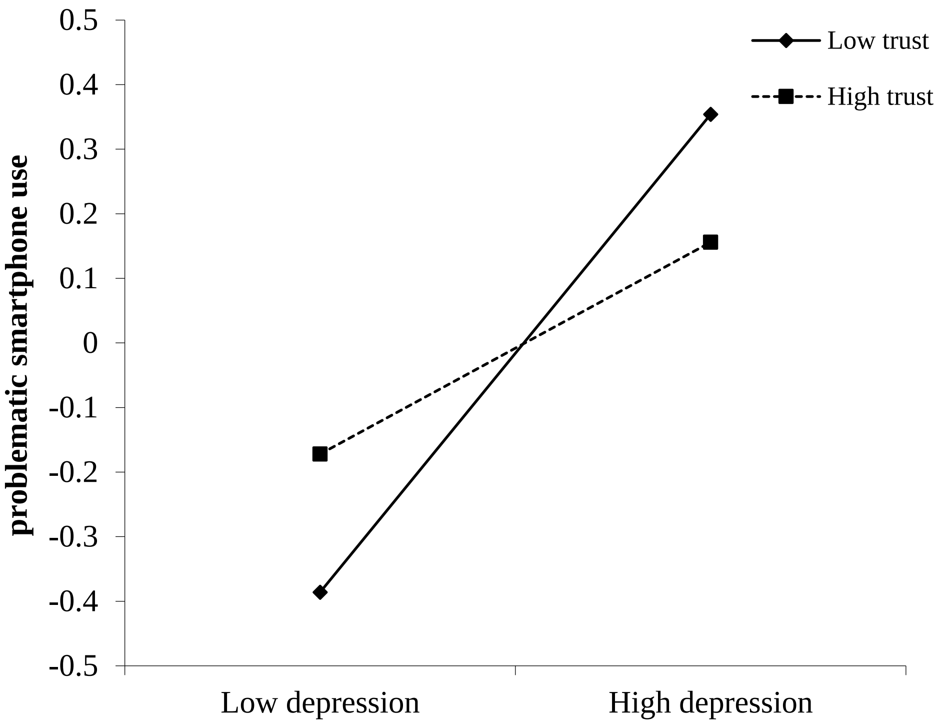

Supplementary Figure 2. Problematic smartphone use (Z-score) as a function of depression and interpersonal trust. Functions are graphed for two levels of interpersonal trust: one standard deviation above the mean and one standard deviation below the mean. Note that the graph is for descriptive purpose only. All inferential analyses maintained the continuous values of depression and trust.
